# Novel Virtual User Models of Mild Cognitive Impairment for Simulating Dementia

**DOI:** 10.1155/2015/358638

**Published:** 2015-08-03

**Authors:** Sofia Segkouli, Ioannis Paliokas, Dimitrios Tzovaras, Thanos Tsakiris, Magda Tsolaki, Charalampos Karagiannidis

**Affiliations:** ^1^Centre for Research and Technology Hellas (CERTH), Information Technologies Institute (ITI), P.O. Box 60361, 6th Charilaou-Thermi, 57001 Thessaloniki, Greece; ^2^Department of Special Education, University of Thessaly, Argonafton & Filellinon Street, 38221 Volos, Greece; ^3^Pan-Hellenic Federation of Alzheimer's disease and Related Disorders, 13 P. Syndika Street, 546 43 Thessaloniki, Greece

## Abstract

Virtual user modeling research has attempted to address critical issues of human-computer interaction (HCI) such as usability and utility through a large number of analytic, usability-oriented approaches as cognitive models in order to provide users with experiences fitting to their specific needs. However, there is demand for more specific modules embodied in cognitive architecture that will detect abnormal cognitive decline across new synthetic task environments. Also, accessibility evaluation of graphical user interfaces (GUIs) requires considerable effort for enhancing ICT products accessibility for older adults. The main aim of this study is to develop and test virtual user models (VUM) simulating mild cognitive impairment (MCI) through novel specific modules, embodied at cognitive models and defined by estimations of cognitive parameters. Well-established MCI detection tests assessed users' cognition, elaborated their ability to perform multitasks, and monitored the performance of infotainment related tasks to provide more accurate simulation results on existing conceptual frameworks and enhanced predictive validity in interfaces' design supported by increased tasks' complexity to capture a more detailed profile of users' capabilities and limitations. The final outcome is a more robust cognitive prediction model, accurately fitted to human data to be used for more reliable interfaces' evaluation through simulation on the basis of virtual models of MCI users.

## 1. Introduction

As people age, they experience alterations in cognitive abilities such as working memory, at attention processes and spatial cognition, especially when confronting new technology [1]. The design of human-computer interfaces' requires careful study of multitasking capability [2] and has to take into account cognitive aging changes on older adults' perception [3]. The line between normal aging and dementia may comprise conditions in which heterogeneous patterns of cognitive impairment may be observed. Indeed, memory disorders with no dementia in the elderly population are frequently reported, and their prevalence varies from 22% to 56% [4].

So far, research efforts have developed cognitive architectures and theories in order to capture the essential representations of cognition. This activity has gradually moved from a focus on the functional capabilities of architectures to the ability to model the details of human behaviour, and, more recently, brain activity [5]. Cognitive models have been used to simulate humans' performing multiple tasks in order to improve the quality and usability of the interface designs [6]. A bunch of models that merge psychology and artificial intelligence termed as cognitive architecture like SOAR [7], ACT [8], and EPIC [9] have been used to simulate human-machine interaction to both explain and predict interaction behaviour. A simplified view of these cognitive architectures is known as the GOMS model [10]. Commonly, models developed using cognitive architectures consider the uncertainty of human behavior in detail but have not been widely adopted for simulating HCI as their use demands a detailed knowledge of psychology [11]. Existing user modeling tools, focusing on inclusive interaction like EASE [12] or CogTool [13], do not yet cover a wide range of users with perceptual, cognitive, and motor disabilities.

The SOAR architecture was created to demonstrate general human-level intelligence and focuses more on high-level functionality than on low-level cognitive fidelity, which makes it less suited to predict people's errors and limitations [14]. EPIC is especially suited for modeling human multimodal and multiple-task performance including sensory-motor processors. The ACT-R theory for simulating and understanding human cognition enabled serious consideration of the cognitive, perceptual, and motor capabilities of the user, to describe interactivity in HCI [15] and provided a reliable cognitive architecture for the development of large-scale, functional, cognitively motivated models. ACT-R implementations may vary in technical details such as time parameters of the user model [16]. For computational reasons, most actual ACT-R implementations use additional submodules as noise generators applied on the output in order to create noisy parameters.

The cognitive module proposed in this paper is based on a modification of the ACT-R implementation model in order to achieve more accurate prediction of the MCI elderly memory changes mirrored at the performance on infotainment tasks. One effective approach could be to intervene in the internal part of the ACT-R cognitive architecture by introducing a dual submodule (one for the declarative memory and one for the procedural) that can affect memory as well as the overall cognitive functionality. The further challenge was to determine the error rates and the extra time needed by MCI virtual user models (VUMs) and compare timing and success rate results with those generated by real MCI users while interacting with interfaces.

*(1.1) Cognitive VUMs Based on ACT-R.* Cognitive models have been used to improve the quality of interface design by applying what is known from psychology to the design of interfaces [6]. ACT-R, EPIC, and SOAR have been employed extensively in psychology and the cognitive sciences to model human behaviour [17]. Moreover, these techniques may be used to explain and predict human-computer interaction. However these cognitive architectures, known as the GOMS (goals, operators, methods, and selection rules) model, are mainly suitable for modeling the optimal (skilled) behaviour of users.

Various user model representations have been proposed from ontology based architectures [18] to XML-based languages [19]. Based on ACT-R cognitive architecture, Serna et al. [20] aimed to model and simulate cognitively impaired people such those suffering from Alzheimer's disease by evaluating performance in the execution of an activity of daily living. They simulated the loss of memory and increase in error for a representative task at kitchen by changing different ACT-R parameters. The technique is interesting but their model still needs rigorous validation through other tasks and user communities.

Biswas et al. [21] developed a simulator to help with the evaluation of assistive interfaces using the CPM_GOMS [10] model to simulate the optimal behavior and a new model based on Markov processes for suboptimal behavior. The CogTool system [22] builds on the existing architecture of GOMS models and ACT-R system in order to provide quantitative prediction on interaction. However, the system simulates expert performance with GOMS modeling support while the ACT-R system helps to simulate novice users. It does not yet seem to be used for users with disability or assistive interaction techniques. The GUIDE user modeling system considers the needs of users with disability and age related impairments. Despite the many conceptual advances in cognitive architecture research and the strength in modeling cognition and general intelligence, there are some open issues that deserve attention. Among their known limitations is that detailed physical, cognitive, and behavioral characteristics cannot be sufficiently represented.

Comparatively little research has been paid on the simulation of memory loss and more rigorous approaches of predictive parameters that concern specific population groups such as the mild cognitive impairment are needed. Virtual user models have commonly been shown to provide insufficient knowledge to the product designers in order to differentiate normal from pathological origins of age-related cognitive changes and provide interface prototypes with enhanced accessibility.

*(1.2) Novel MCI Virtual User Models.* In our approach, ACT-R has been chosen as the theoretical framework as it is the most representative example of cognitive modeling architecture being pushed to its limits by large-scale modeling and used to develop specific models. ACT-R comprises a series of modules which serve to represent different information such as goals and declarative memory. ACT-R can model characteristic errors of human performance; however, the work in cognitive simulation is in progress and in continuous update to reflect subtle changes of cognition and behavior. The specific challenge was to extend capabilities and functionality of already existing cognitive mechanisms. Therefore the proposed dual submodule could be viewed as a shift in specific cognitive representations. Moreover different cognitive modules could be plugged in for more accurate evaluation purposes.

Initially, our main challenge was to enhance the cognitive realism of computer generated models and afterwards provide a second generation of VUMs on the basis of a novel submodule that will reflect representative and subtle cognitive changes of elderly and people with memory decline as an early onset in mild cognitive impairments. MCI is the transitional stage between normal ageing and dementia. More specifically, amnestic mild cognitive impairment (aMCI) is characterised by memory complaints, so the simulated memory of the VUM had to be affected. Moreover, verbal fluency ability is affected in a MCI and thus some extra delays in communication tasks would give VUM results closer to those of the actual users [23].

In order to describe a set of users with specific disabilities we instantiated a generic VUM (GVUM), representing the specific group of elderly and MCI population. The general concept of the generic user model has been defined by the Veritas project VUMs [24]. In this experiment, as in previous studies [25], these instances of the GVUMs were intended to be used for simulation purposes to test user interfaces against various disabilities. In order to form and evaluate the proposed virtual user models we have been based on high level languages for describing cognitive models and simulation frameworks supporting these languages. More specifically, our virtual user models were formed by separating user models from task models according to the UsiXML Language [26].

Our simulator, based on well-known literature findings [25, 27], like the Hick-Hyman law for cognitive information capacity [28, 29] and Fitts' law for human movement [30] became capable of predicting the number of interaction events within infotainment scenarios and the time needed to complete them [31]. Hick's law states that the time between a stimulus and the user's response is increasing by the number of available choices at any given time. Users, when facing a decision making problem, need more time to react as their perceived alternatives increase. In a GUI control element, for instance, a long drop-down list requires more time to pick the wished choice. The time is calculated in the beginning of the user's reaction in order not to be confused with motor abilities. Hick's law was not used in equally probable choices; the following formula was used to take into account the entropy of the decision:

(1) $$\begin{array}{c} H=\overset{n}{\underset{i}{\sum }}p_{i}\log_{2}\left(\frac{1}{p_{i}}+1\right), \end{array}$$

where *p*_*i*_ is the probability of the *i*th alternative (information theoretic entropy). Fitt's law is a well-established model which describes the time needed for a human movement from an initial state to a target based on the (a) Euclidean distance to the target and (b) the size of the target. In simple words, this model is used to predict pointing actions either by a physical touch or a virtual one, like in cursor pointing using a mouse device. The predicted time is given by the following equation:

(2) $$\begin{array}{c} T=a+b\;\log_{2}\frac{2D}{W}, \end{array}$$

where: *T*: time needed to complete the task, *a*, *b*: model parameters, *D*: distance from the current cursor position to the center of the target, and *W*: width of the target along the axis of motion.

To overcome the limitation of screen sizes, the size of the target *W* is considered very large (infinite) in case of targets located at the edge of the screen. Task analysis and prediction of real user's performance (durations) have been used in the past with good prediction accuracy over predefined tasks [32].

**Figure 1:**
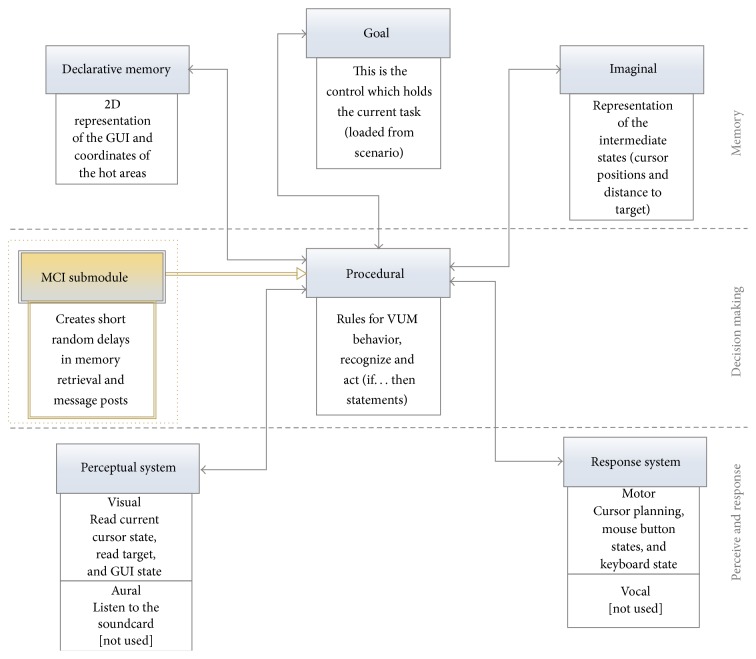
The revised cognitive model of the VUMs (extended ACT-R model).

This study investigated how subtle changes in cognition could affect GUI interaction for elderly and MCI groups. Cognitive functioning data correlating with memory capacity have been considered to be of critical importance to be encoded into VUMs that would be assessed through simulations over GUIs.  Figure 1 briefly describes the submodules of the new ACT-R approach. The first line contains memory components which are being affected by cognitive impairments. The control component located at the center (procedural) orchestrates all others by exchanging messages. Located at the bottom are the perceptual system possibly affected by visual and/or aural impairments and the response system where the motion planning of the cursor is generated, along with the button clicks and the key strokes (motor). The extension to the existing architecture is the MCI modeling submodule which temporarily modifies the random rules, memory retrievals, and messages exchanged between other parts of the VUM, to simulate the cognitive decline. An internal interface has been designed for this submodule. For compatibility reasons the functionality of the submodules has been implemented in the simulator with keeping the VUMs structures unaffected.

The modification of the memory-related features of the VUM (mostly declarative memory and current goal) is simulated by inserting additional time delays on the performance of the elderly VUM. Thus, according to the current approach, the time to complete a given scenario by the MCI cognitively impaired VUMs is defined by

- the human movement model known as Fitt's law mounted in the motor sector of the response system,
- Hick's law which describes the time needed by the VUM to make a choice among many interaction controls on a given interface based on its current goal [28],
- the new additional time delay inserted in the memory modules caused by the MCI and being scaled according to the assumed Montreal Cognitive Assessment test (MoCa) [33] and the Boston Naming Test (BNT) [34] results,
- other delays issues to the ACT-R itself, the hypothetical age of the VUM, or the existence of physical impairments.

Summarizing, the proposed MCI submodule is affecting the error rate and the execution time of tasks. Those two parameters were trained using tests with real users that are reported in the next section.

## 2. General Objectives

Computer and information technologies' use could improve the quality of life of older people. However, successful use of technology by the elderly is limited by age-related cognitive and perceptual changes. Designers have to use as an index not generally age but concrete cognitive, perceptual, and behavioural changes to identify early indicators of dementia and customize the entire process of design accordingly [1]. Existing design practices have provided a few accessibility features for elderly or disabled users, mainly covering motor, vision, and hearing impairments. Unfortunately, up to now accessibility guidelines do not systematically consider users with perceptual and cognitive abilities [21].

Within this research work, we undertook a multifacet study, focusing on robust cognitive prediction models for mild cognitive impairment within the scope of graphical users' interfaces accessibility and evaluation. The main aim is to introduce novel virtual user models with enhanced predictive validity in mental processes that will be utilised for accurate simulation results in interface design.

Cognitive-based evaluation of interface accessibility and evaluation are proposed as interface design support towards more inclusive graphical user interfaces. However, user interface evaluation is also the means to arrive at the optimization of the proposed novel cognitive models. Our approach is to initially research the relationship between some design variables and the mental processes required to perform successfully some indicated scenarios and then utilise the effectiveness of user interfaces to optimize the MCI VUMs. The main vision of the proposed research is approached through the synthesis of the following objectives.

- Model elderly with mild cognitive impairment for an in-depth analysis of their perceptual and cognitive abilities.
- Evaluate cognitive decline through GUI accessibility assessment.
- Utilize the effectiveness of user interfaces to optimize MCI VUMs.

To that end, we organized our work in a set of 4 studies, each one aiming to provide a specific set of qualitative and quantitative results in order to arrive at the aforementioned objectives. These studies, as well as their methodologies and results, are detailed in the following sections.

## 3. Study 1: Preliminary Study with Real Subjects

### 3.1. Introduction to Study 1

With an ever-increasing proportion of the total elderly population suffering from MCI symptoms, GUI designers need to deliver ICT products which have been previously tested for their accessibility features. According to our approach, simulation testing can make this possible to a certain extent using VUMs instead of real users. To train the MCI cognitive VUMs, that is, to stabilize the cognitive-impaired VUM parameters and regulate their simulating performance, we first needed to assess the MCI symptoms of real users and then examine these users' performance (extra time needed to complete tasks and a higher error rate). To achieve this, initially real users have been recruited to perform well-established screening tests for cognitive evaluation.

### 3.2. Materials and Methods of Study 1

Among various MCI screening tests that are already applied in day-care organizations, the short version of BNT screening test (30BNT) was chosen as one of the most well-established MCI tests. The MoCa test, supplementary to BNT, was also chosen due to its verbal fluency part so as to test users' verbal ability and strengthen their profiles. A computer-based version of the aforementioned tests was implemented and its validity has been checked against the paper-and-pencil versions of the tests.

The test had five discrete steps: (a) introduction to the scopes of the test by the pilot test supervisor (5 min), (b) initialization of hardware and software (3 min), (c) creating new user profile (1 min), (d) performing a demo example for each test (30 sec), and (e) actual test and log file creation (20 min). During the screening tests, the cognitively impaired elderly, as well as all other elderly and young participants, did not have to navigate themselves in the screening application. Instead, a member of the research personnel (RP) was navigating for them; from the participant's log files a constant time delay factor (psychomotor noise) was removed to correct the timestamps. A simple reaction time (SRT) was taken as a constant calculated from Hick-Hyman law [28, 29].

Considering the right and wrong answers of the elderly as equally probable choices, the RP had to press the “correct answer” button or press the spacebar to start writing the user's own word. Each RP, who was responsible for pilot tests, performed ten (*N* = 10) tries on a reaction time test to measure the response times for keyboard presses and mouse clicks. The average response times were removed from the time-stamped answers of all elderly testers who were recorded by this RP. Regarding the BNT test, the subjects who were unable to provide the correct name of the depicted object within 20 s were given a semantic cue and if they were still unable to give the answer after a further 20 sec, they were given a verbal cue. The 20 sec threshold for giving help was given by the institution responsible for taking the paper-based BNT test.

**Figure 2:**
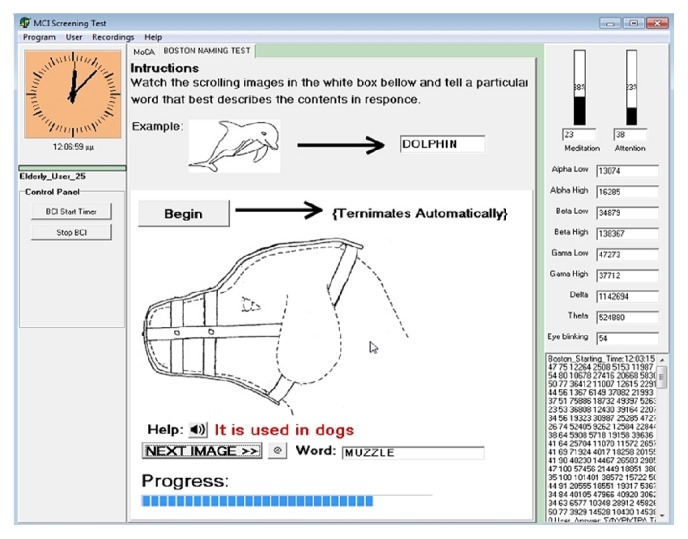
The computer-based BNT Interface.

Finally, the screening assessment produced log files which contained the time-stamped responses of the participants. A mass log reader tool was used later for data extraction. The screening test itself, as well as the log files reader and other supplementary software tools, were engineered with Delphi and C++ programming languages. A screenshot of the computer-based MCI screening test interface can be seen in Figure 2.

MCI screening tests were performed with real users, constituted by three groups: (a) young people, (b) healthy elderly, and (c) MCI users (aMCI). Recruitment criteria for MCI users included at least 55 years of age, being self-handlers, having basic computer skills and fluency in Greek language (checked by phone interview). Twenty-five (*N* = 25) elderly persons of different ages from 55 to 78 (*M* = 65.56, SD = 5.89) composed the testing group. Approximately 40% (*N* = 10) of the elderly were MCI positives, nondemented. The control group consisted of eleven (*N* = 11) young expert computer users, 26.27 years old in average (SD = 1.95).

### 3.3. Results of Study 1

The data produced by the computer-based version of the MCI screening tests showed significantly strong correlation with those produced by the institutionalized hard-copy versions of the 30BNT and MoCa. This initial step was necessary to make the computer-based versions of the MCI screening tests a valid reference for the following steps. To be noted that the follow-up time of BNT test was six (6) months later in average for most participants and less than one (1) year for all people taking part in this study.

The linguistic MoCa results were found normally distributed (Shapiro-Wilk test), in contrast with BNT results which were not, since results were significantly negatively skewed. Linguistic MoCa results for healthy elderly (*M* = 13.80, SD = 4.94) were found significantly different (*t* = 2.317, *P* < 0.05) than those having MCI (*M* = 9.10, SD = 4.84). The Shapiro-Wilk test showed significant deviance from normality for BNT results, so nonparametric test was used. The Mann-Whitney* U* test gave *P* < 0.05 for the elderly group. So, among elderly users, those having MCI (*M* = 21.78, SD = 5.40) had significantly different BNT results than the healthy ones (*M* = 25.90, SD = 1.97) as expected. Regarding the age variable, the independent samples test showed that, as expected, no significant differences were found on linguistic MoCa results between the elderly users and the young group (*P* > 0.05) [35]. Similar results were found for BNT scores: a Kruskal-Wallis test confirmed that age does not make the difference (*P* > 0.05).

The Mann-Whitney* U* test for the duration of the computer-based BNT test, a variable that was found not normally distributed, showed that the time needed by participants to complete the BNT is not an efficient separator for MCI users (*P* > 0.05) and only the right-responses are.  Table 1 presents the results per young, well-being elderly and MCI groups. Research results confirm that among young and healthy elderly the decline in lexical retrieval is not notable [35]. Finally, variables of the linguistic MoCa and the BNT present a notable correlation (*r* = 0.395, *P* ≤ 0.05), though further analysis revealed different qualities among them. Furthermore, statistical analysis reported good cohesion and separation ability of the linguistic MoCa and BNT clustering model with a score of about ≈ 0.6.

### 3.4. Brief Discussion of Study 1 Results

The screening procedure through MoCa and BNT tests serves as a starting point to the whole methodology of the current research work. Initially, the MCI symptoms of real users had to be assessed as evidence of cognitive decline. This evidence was needed to formulate the quantitative data which will be later related to users' performance while performing interface tasks. Thus, results from MoCa and BNT screening tests assessed users' cognitive and perceptual factors that encompass also the mental state behind response time and general behavioral performance that will be resulted from the following study.

## 4. Study 2: Simulating Tasks with Cognitive VUMs

### 4.1. Introduction to Study 2

Our proposed VUMs were trained based on the data obtained from the afore-described screening tests. The trained MCI cognitive VUMs were then used for the simulation of tasks execution in human-computer interaction with graphical user interfaces (GUI). VUMs in this paper are named according to the cognitive impairment they simulate and the strength (percentile of the total population) of the impairment they correspond to. For example, the “75MCI” VUM is a virtual user model which can simulate the behavior of the 75% of the cognitive impaired elderly population, which is calculated by creating a Gaussian distribution from the obtained data and taking the 75th percentile (i.e., the distribution's value, below which 75% of the observations may be found). The alphanumeric suffix describes the impairment and the numeric prefix indicates the strength or the earliest symptomatic stage of MCI.

**Figure 3:**
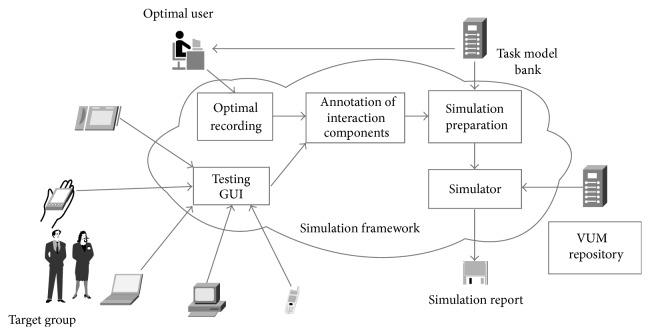
The basic components and data flow of the simulation framework.

To obtain a reference for comparing the performance of the virtual users (i.e., trained VUMs) to the performance of real users, initially, the testing GUI application is initiated in the host device (PC, laptop, smartphone, PDA, and tablet) and the optimal user (OpUs), who is a nonimpaired real young user with expert computer handling abilities and sufficient knowledge of the testing interface, is asked to perform a series of tasks. Those groups of tasks, called scenarios, are described by the task model specification (an XSD schema) and are stored in the task model bank. The optimal recording is critical as it provides a proper reference (ground truth) for comparing the performance of VUMs to the one of real users at later stages. Before any simulation preparation, GUI designers need to define the activities they want to test for accessibility. This means that a formal description of the series of actions in order to arrive at a given result must be produced. To proceed to the actual experimental phase, we introduce “scenario files,” which describe structured tasks used in order to describe the expected, by either real user or VUM, activity during the computer-based experiment. Those files initially describe tasks in an abstract format (e.g., locate another user in the Metaverse), but using a scenario editor tool we developed that they are matched to actions performed on the actual user interface by annotating which areas of the testing interface correspond to active GUI elements in order to match the tasks with the related GUI elements. Eventually, the annotated interface is used in making connections between the interaction events and the tasks described in the scenario (simulation preparation).  Figure 3 gives an overview of the simulation framework and the basic data flow between the main components.

During the simulation phase, initially we constructed three representative cognitive models: (a) elderly, (b) MCI, and (c) young users' group. These models were thereafter used to simulate scenarios similar to the ones performed by respective real users, toward determining and validating the comparison capacity of the system. Task validation was expanded in iterative trials with slightly different scenarios structured as sets of tasks. Those tasks were randomised so as to mitigate the learning effect. The results of the initial validated cognitive tasks were correlated and analysed with regard to the overall duration and the number of the low-level (LL) interaction events related to the input devices produced during the experiment.

The LL events included keystrokes cursor *X*-*Y* movement on the screen and mouse button clicks all hooked by a custom software tool from the lower OS layers (operating system messaging system). According to our simulation approach, the number of interaction events worked as an error rate indicator. Additionally, the “soft fail” concept was introduced to allow VUMs to retry the failed tasks in simulation time. Correspondingly, the “hard fail” concept terminates the simulation at the very first fail-point. In “soft fails” the VUMs have the chance to retry on failed tasks up to the limit of 200% of the OpUs time. This limitation allowed VUMs to repeat internal trial-and-error cycles without exceeding the doubled OpUs duration. We introduced this technique of VUMs to retry on failed tasks in order to prevent unsuccessfully completed tasks by a constrained VUM in infinite time (endless loop).

Finally, experimental results were exported in simulation reports which contained detailed information about the VUM's performance. Simulation reports were structured with respect to an XSD schema definition to formally describe measured elements as extensible markup language (XML) documents. A short human-readable description on the findings, including statistics on error rates, durations, number, and type of OS interaction events was included in the header of the simulation reports in order to allow results comparison with the performance of the real users (saved in log files). Real users performed in an external-to-the-simulator software environment, namely, the MCI screening tool and the real user's logging approach.

The accessibility assessment approach using VUMs proposed herein could effectively describe memory decline in mild cognitive impairment. The main quantitative measures were (a) the number of interaction events produced by the user during the scenario execution and (b) the time needed to complete the scenario (duration). Infotainment results were initially checked for correlation with age and MCI results, to construct a prediction model upon statistical methods using SPSS v.19. Moreover, the proposed cognitive models, based on the new dual cognitive module, were evaluated over whether they are better predictors of the time that actual MCI patients need to complete the tasks than the previously used cognitive architecture, without the new MCI submodule.

### 4.2. Methods of Study 2

**Figure 4:**
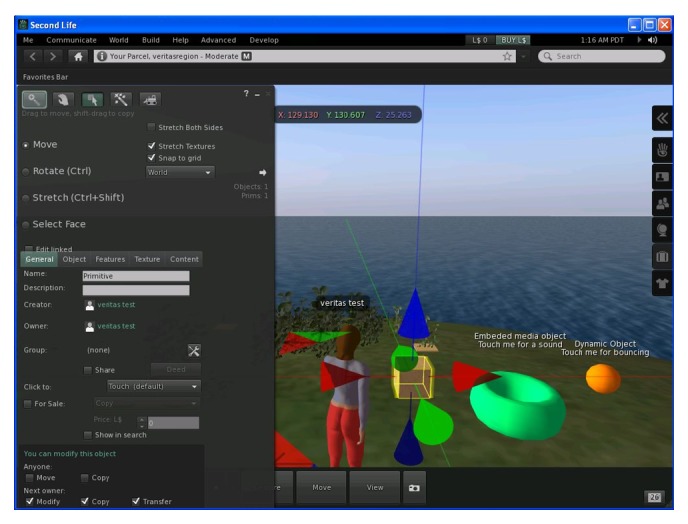
The Metaverse viewer used here as the testing interface.

Pilot studies with elderly and MCI users took place on September 2013. The second life (SL) viewer of (Figure 4) was used to allow participants interact with the Metaverse and other distant visitors. The special version of the SL application used in the infotainment tests was developed using the OpensSim server platform and the source code version of the official SecondLife viewer application. This way, a private and fully controlled by the authors' team SL Metaverse environment—not visible from other SL visitors—was made available for subjects (real users and VUMs). It is worth noting that the capability to control the custom Metaverse refers not only to the features of the interface, but to the content of the immersive environment as well (2D and 3D content and functionality).

The infotainment tests were completed in the same day as the computer-based MCI screening test (Figure 4). In tests' iteration the scenarios were presented at a slightly different manner in order to eliminate the learning effect. However, the difficulty level of tasks has been maintained at both pilots in order to be comparable.

After performing the MCI screening tests, participants were entering a spacious demo room reserved for the pilot tests, usually in pairs. To ensure participants feel confident in using the Metaverse interface, after a demonstrating execution of all scenarios by the RP, the elderly participants were asked to follow. Thirteen relatively easy scenarios of common infotainment tasks were created for the test purposes. Some examples of scenarios are (Table 2): “enter the Metaverse” which is the user authentication process (S1), “change outfit in avatar's appearance” (S2), “create and manipulate 3D objects” (S4–S7), “navigate avatar” (S8-S9), “interact with objects” (S10-S11) and “interact with other visitors” (S12-S13). The length of each scenario, represented by the “number of tasks” indicates how many tasks must be completed by the user successfully to achieve the scenario goals.

### 4.3. Results of Study 2

The infotainment findings indicated that the number of interaction events, which also expresses the error rate due to the “hard error” and “soft error” concept, is a not a strong separator for the elderly and the MCI groups, so it was gradually repealed.  Table 3 presents in detail the results of the infotainment pilot test organized per scenario for the two testing groups (elderly and MCI).

The research hypothesis here is “*if some portion of the population has cognitive impairments which affect memory, then for those who failed in the MCI screening test, it is expected that their scores in the related infotainment scenarios will be lower than for others.*” It is critical that not all the scenarios are equally interesting for the dual cognitive module development. For example, the scenarios where mostly motor, visual, and acoustic abilities are needed for their successful performance were intact for the MCI users. Indeed, among the available GUI scenarios, those which require more complex mental processes proved to be the appropriate for the distinction between MCI and healthy elderly users. Thus, in this study, emphasis is given on those scenarios which are primarily affected by memory and decision making decline.

Having the scores expressed as percentage differences of the OpUs performance, healthy elderly perform the infotainment scenarios in the 432.94% of the OpUs time and they produce the 50.77% of the OpUs events. Similarly, the results for the MCI users are 446.48% of the OpUs time and 56.20% of the OpUs events. Although the average values show that MCI users need more time and more keyboard and mouse clicks to perform the same scenarios; this extra effort may not be significant.

After normality tests, all variables related to scenario duration and events were tested for equality of means in the two testing groups (healthy elderly users and MCI users). For healthy elderly, the time needed to complete the first scenario (S1: *M* = 59.53, SD = 21.85) and the last one (*M* = 83.43, SD = 25.05) was found to be significantly different than the duration of the first (*M* = 87.00, SD = 32.34) and the last scenarios (*M* = 121.53, SD = 45.42) of those having MCI (S1: *t* = −2.21, *P* < 0.05 and S13: *t* = −2.34, *P* < 0.05). Also, this difference was found at a significant effect size: *r* = 0.445 for S1 and *r* = 0.461 for S13. For all other scenario durations, the null hypothesis for equity of means was not rejected.

Regarding the other test metric, the number of interaction events (keystrokes and mouse clicks produced during the experiments), the null hypothesis for equity of means was not rejected (*P* > 0.05) in all tests. Concerning the number of interaction events, results indicate no difference between the two target groups. The same result was extracted from the test (Mann-Whitney* U* test) between the institutionalized and noninstitutionalized MCI.

### 4.4. Brief Discussion of Study 2 Results

**Figure 5:**
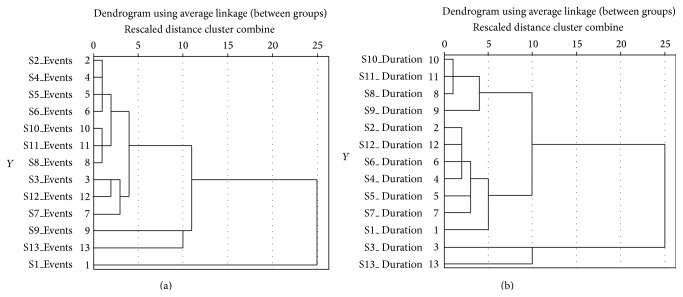
Two dendrograms as result examples which represent the variables of the number of interaction events (a) and time in seconds (b) using average linkage between groups.

The duration and the number of events for each scenario are not always following each other's fluctuations. Strong correlations between the duration and the number of interaction events were found only on scenarios S2 (*r* = 0.730, *P* < 0.001), S5 (*r* = 0.669, *P* < 0.05), S6 (*r* = 0.525, *P* < 0.05), S9 (*r* = 0.477, *P* < 0.05), and S11 (*r* = 0.859, *P* < 0.001). In Figure 5 two representative dendrograms of the duration and events variables are presented as the result of the hierarchical variable clustering. The vertical axis represents the number of interaction events (Figure 5(a)) and the overall duration (Figure 5(b)).

Following the results of the running data through the average linkage between groups, it is obvious that the two memory-related scenarios S1 and S13, as well as the two information orientation scenarios S3 and S13 are outliers and are fused in arbitrarily at much higher distances than others. The main aim was to extend all the knowledge acquired by the statistical analysis of the infotainment data in order to optimise the VUMs performance towards a second generation of VUMs, especially designed and tested for the selected MCI group.

The following section describes how the measures of the experiment were fitted to a regression equation in order to develop a performance prediction model for the second generation of VUMs. The expecting generation of VUMs, optimized based on the findings of study 2, summarizes all our expectations regarding the prediction of real MCI user's performance when working on interface designs.

## 5. Study 3: Optimization of VUMs

### 5.1. Introduction to Study 3

The currently presented effort is about the optimization of VUMs based on real users' data and the cognitive architecture proposed by the ACT-R model. The ultimate goal was to create robust VUM cognitive models as an internal mental representations of elderly users that will constitute their exact cognitive profile. In a first level the insertion of the MCI submodule as a perceptual module targeted at better estimations for early dementia prodromes. The new challenge was to determine the error rates and the extra time needed by MCI-VUMs and compare timing and success rate results with those generated by real MCI users. In a second level, a more detailed representation of the VUM cognitive model was used in interface accessibility assessment in a simulator. The results extracted from the infotainment test were used to compute which cognitive parameters should be updated.

### 5.2. Methods of Study 3

A regression equation was used to find out what relationship, if any, exists between the durations of performed—by real users—infotainment tasks and MCI screening test results given by the same subjects. As long as the predicted VUM's scores, as outcomes of the simulation tests, can fit to a regression equation, we can remodel the cognitive submodule of the existing VUMs for future experiments (optimized VUM).

This section explains the results of the forward selection procedure we followed as a strategy to choose the order of the approximate polynomial which calculates the scenario durations given the MCI screening scores. Using the BNT score as the independent variable, we successively fitted the models in increasing order and tested the significance of regression coefficients at each step of model fitting.

At first we assumed linearity as a starting point for calculating an equation which minimizes the distance between the fitted line and the experiment measure points (scenario durations). The linear regression equation was considered statistically significant (*P* < 0.05) and it gave a nice fit in our experimental results. Next, following our forward selection strategy, we upgraded our model to become a second-order model by trying to keep the statistical significance of the highest order term in the range of accepted values (<0.05) and at the same time to increase the *R*-squared (*R* ^2^) statistic (coefficient of determination). Calculated as the percentage of the response variable variation that is explained by our linear model, the *R*-squared statistic was found to be equal to 0.322. Keeping this value for future reference, the quadratic model can be expressed as

(3) $$\begin{array}{c} Y=X^{2}b_{2}+Xb_{1}+c, \end{array}$$

where *Y*: duration of scenario, *X*: MCI screening score (BNT), and *c*, *b*_1_, *b*_2_ are calculated by the equation system.

And for *n* being the size of the data

(4) $$\begin{array}{c} \sum Y_{i}=nc+b_{1}\sum X_{i}+b_{2}\sum X_{i}^{2}, \end{array}$$

(5) $$\begin{array}{c} \sum {X_{i}Y}_{i}=c\sum X_{i}+b_{1}\sum X_{i}^{2}+b_{2}\sum X_{i}^{3}, \end{array}$$

(6) $$\begin{array}{c} \sum {X_{i}^{2}Y}_{i}=c\sum X_{i}^{2}+b_{1}\sum X_{i}^{3}+b_{2}\sum X_{i}^{4}. \end{array}$$

The above method was used to calculate the expected duration of the scenarios when MCI users took part in the experiment. The final simulation report gives as predicted duration

(7) $$\begin{array}{c} D_{i}^{\prime }=D_{i}+C_{i}^{\prime }, \end{array}$$

where *D*_*i*_′: duration of scenario *i* for a VUM with 50% MCI, *D*_*i*_: duration of scenario *i*, for a healthy elderly VUM, and *C*_*i*_′: constant coefficient.

As an example, given the duration of scenario 1 for a healthy elderly, the coefficients of 3 for calculating the duration of the scenario using a 50MCI VUM were found *b*_1_ = 41034.988, *b*_2_ = −979.606, and *c* = −399951.739.

In order to keep the simple rule that cognitive decline (e.g., MCI) adds extra time to the duration of scenarios, *C*_*i*_′ in 7 must be always greater than zero (nonnegative). So, the standard coefficient *c*, as calculated before, subtracts *D*_*i*_ from the result of 3 in order to express the amount of time added to the healthy elderly as a result of the cognitive decline.

The quadratic model gave an accepted statistical significance (*P* < 0.05) and moreover it offered a significantly higher *R*-square (*R* ^2^ = 0.459) than the linear model (*R* ^2^ = 0.322). By carrying on the model upgrading procedure, the next polynomial regression model (the cubic) was found not much better (*R* ^2^ = 0.456, *P* < 0.05) than the previous one and thus we did not upgraded our model more. The second-order model was more preferable due to its simplicity, so the quadratic effect parameter remained as the highest order term in our prediction model. To be noted is that other models, like the logarithmic, power, and exponential, were used to give no better results than the quadratic model.

The prediction models used for calculating the more accurate task durations of the MCI VUMs were incorporated into the GUI simulator to achieve optimized simulation results. Thus, the simulation reports produced on the later stages of the experiment contained more accurate results and were better fitted to the GUI designer's decision making. The optimized VUM behaviour was now both visually observed during simulation and imprinted in experiment reports. This was achieved by taking into account the updated parameter values which were responsible for the control of the simulation time flow. The optimized time management regulation mechanism had a direct effect on the task completion times of the MCI VUMs, while in VUMs free of abnormal cognitive decline, the GUI simulation functionality remained unaffected. To be noted is that the optimized functionality of the MCI VUMs refers only to the time delays related to the cognitive decline and not the other kinds of time regulators (like in the motor submodule, e.g.).

The optimized version used the same motor, vision, and perception submodules as in any other case. Only when an MCI VUM was present in a simulation scenario task, the simulator adjusted its time management functionality according to the quadratic prediction model inserted into the optimized VUM. Practically, this was implemented as series of time delays between the task sequence calls. The rate and duration of these delays were corresponding to the degree of the cognitive decline (expressed as a “hypothetical BNT test” result). Finally, when the experimental simulation tasks were performed, the visually perceived difference between a healthy elderly and an MCI VUM (macroscopical difference) was like an unexpected or unjustified delay in the cursor motion planning. This distortion on cursor movement was due to the simulated “forgetfulness” of the VUMs which had lost control over their performed tasks, although their tasks were simply noted in the loaded scenario file.

Another important time regulator of the optimized model is the time needed by the VUM to select one option in the interface instead of another, when many options were present at the same time. This is quite similar to the common question “Which button does the job, this one or some other?” The simulator takes into account the Hick-Hyman law as already described, but not the human processing speed that may be affected because of the intelligence quotient (IQ) of the VUMs [36]. Real users were not asked to give an IQ test before or after the test and so the IQ was not taken into account by the optimized VUM model.

The outcome to this point is a more detailed representation of the VUM cognitive model for use in interface accessibility assessment in a simulator. The results extracted from the infotainment test were used to compute which cognitive parameters should be updated and by which factor in order to eliminate the differences between the scores of VUMs and the real elderly users.

### 5.3. Results of Study 3

The new cognitive architecture, inserted into the cognitive-aware VUM was evaluated against the old VUM generation to measure the benefit from the adoption of the new cognitive model. As an example, in Table 4, a comparison between the time scores of the real MCI users, the 50Elderly (old VUM) and the 50MCI (new VUM) in seconds are presented. Data were extracted by simulating the “Enter the Metaverse” scenario (S1) and the optimal user's performance has been subtracted.

Practically, the optimization of the cognitive models was proved by using statistical methods over a set of experimental time and score recordings. A number of regression models have been used for prediction purposes as it was previously described. The lower and upper bounds of the 95% confidence interval for each of the coefficients of the regression equation were used to produce the range of the percentage MCI VUMs, starting from the barely 1MCI up to the maximum 100MCI. As an example when the OpUs needs 15.35 sec to complete the user authentication scenario (S1), the healthy elderly need 59.53 sec on average as seen in the infotainment pilot test, and the average MCI user needs 87 sec for the same task.

The 50Elderly VUM needed 17.30 sec, which is far away from the actual user because a VUM does not take into account common typing mistakes made by the average user or momentary lapse of memory (e.g., forgetting one's password). This exemplary performance is not convincing because it is closer to the OpUs than to the elderly or the MCI users. The only time-penalty paid above the OpUs duration is an age-related delay inserted in the motor module (cursor moves) to justify the difference (1.95 sec).

The 50MCI on the other hand, created with BNT = 22 in cognitive scores, needed 88.21 sec that is 98% similar to the actual MCI user's score, in contrast with the old 50Elderly VUM which needed only the 19% of the actual time score.

### 5.4. Brief Discussion of Study 3 Results

The purpose of this study was the optimization of VUMs performance that was made possible after a set of testing scenarios, on actual elderly and MCI users, to fit better the performance of the real persons. The infotainment application area was selected as representative of typical computer-based environments, encompassing from 2D GUI elements to immersive interactivity and social communication with other distant users.

The duration of the scenarios which require strong memory (remember username and password in S1, the sequence of the dialog boxes to open in S13) was found to be affected by the existence of MCI. The same can be generalized for “deep GUIs” which require that users follow long point and click routes to master the application, even if the application itself has a good learning curve. The number of interaction events produced during testing, which hides the success and failure rates of mouse clicks and keyboard strokes, was proven a weak predictor, probably because of the scenario directions themselves: the fact that the elderly and MCI testers were given directions of how to perform the scenarios resulted in inadvertently correcting errors related to the decision-making. A nonsupervised pilot test with real users based on a set of scenarios clearly targeted to the memory-judgment dipole, in which only the goal is shared with the participants, would be a more sensitive instrument to detect time delays and interaction patterns in MCI populations.

Following a deterministic approach, the VUM scores of every previous test are used to predict more accurately the behavioural and cognitive alterations of actual end users by time. This approach allows interface designers to run accessibility tests on their new designs at early development phases without the need to recruit real impaired persons.

The new cognitive module, the development of which was based on the total scenario duration prediction model (quadratic model), worked as expected and produced timing scores closer to the actual MCI users than the previous model. Bearing in mind that the achieved quality in the prediction model development is characterized by the coefficient of determination *R* ^2^, in the field of human behaviour, such as the infotainment experiment, it is entirely expected that *R*-square values will be lower than 25% [37, 38]. The performance of human subjects in complex tasks over complex user interfaces is simply harder to predict than in other research fields. Thus, the resulting value of 45.9% for *R*-square satisfies our research expectations. From the VUM optimization point of view, this was a key-point result because it let us draw the important conclusion that the MCI scores of BNT can explain almost the half of the variability of the response data around its mean.

## 6. Study 4: Accessibility Assessment with Optimized VUMs

### 6.1. Introduction to Study 4

**Figure 6:**
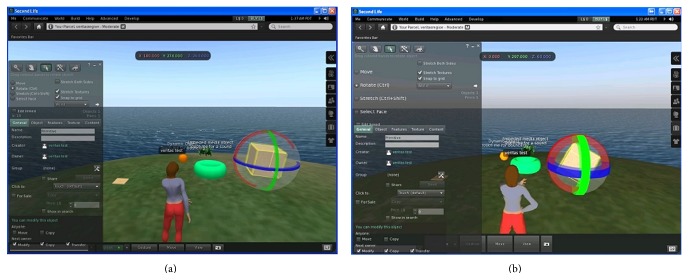
The original Metaverse interface (a) and the new design (b).

This simulation test using the new VUMs based upon the optimized GUI aimed at supporting more tangible graphical user interfaces and directed the Metaverse developers to redesign the user interface according to the needs of all potential users, including those having MCI (Figure 6). Towards VUM optimization according to the needs of MCI users, some dialog boxes have been simplified enough to minimize the cognitive load, while pictograms were preferred for triggering common tasks and thus eliminating memory retrieval.

### 6.2. Methods of Study 4

The new interface was tested by a new group of VUMs, starting from 50MCI and reaching 85MCI. Instead of creating a completely new VUM, the elderly VUM was modified in such a way that an architectural extension (MCI submodule) is affecting its performance in the same way MCI is affecting humans. The duration predictions of the optimized VUM were sliding in the range indicated by the lower and upper bounds of the model coefficients as expected. Above the 90MCI threshold, the interface was considered by its creators as not capable of being redesigned without losing its basic functionality.

### 6.3. Results of Study 4

The test performed following the same protocol on the updated interface yielded better results, namely, reduced times needed to complete the scenarios in average by 14.09%. In the memory-demanding scenarios (S1 and S13), which are of particular interest for MCI users, the elderly reduced the user authentication time to 57 sec (SD = 23.36) from 59 sec (SD = 21.85), but MCI users reduced to 65 sec (SD = 34) from 87 sec (SD = 31.54).

### 6.4. Brief Discussion of Study 4 Results

Accessibility assessment through the optimized VUMs has practically proved that inserting the novel MCI cognitive submodule in the overall VUM architecture provides the potential that interface designers can make tests on the simulator for decision making on interface redesign. The results were proven valuable as the new accessible interface design reduced the memory load in memory demanding tasks and also the cognitive load in decision making tasks. The new generation of VUMs, the cognition-enabled ones, responded in a human-like way pointing to the interface designers which design feature changes were positives according to the accessibility rules.

## 7. General Discussion of All Studies

The simulator used to perform experiments through VUMs was developed to be results-oriented. The ultimate goal was to enhance interfaces' quality in order to support designers performing realistic simulations. On the other hand, the ACT-R model, in which the structure of our VUMs was based, was initially proposed as a meaning-oriented structure for general user modeling use. The multiple studies and experiments we performed targeted the development of a novel cognitive model customized to MCI users as an extension to existing cognitive models.

Each study has contributed in its own way in the overall effort to simulate dementia for interface accessibility assessment. The first study provided the necessary quantitative data to describe the medical state of the participants on the basis of well-established MCI screening tests. The second study has served as a systematic semiautomated observation of MCI and controls over a period of time, during task oriented scenarios. Among the performed tasks, those pointing on memory and decision making processes were of particular importance for the target audience of patients suffering from dementia. The third study indicated the relation between the performed timing scores and the state of cognition. From the results we gathered, the model which best described in statistical importance the relation of user's performance and cognitive decline associated with MCI was a quadratic model. Using these outcomes, we optimized our models according to the results of the previous study checking also the consistency with the simulator requirements. The cognitive submodule in particular, which was inserted into the existing virtual user architecture introducing a new generation of cognitive-aware VUMs, is capable of performing the same tasks as real users in a more realistic manner. The same testbed (Metaverse environment) was used to evaluate the expected benefits on VUM's quality, as a matter of prediction ability. Indeed, the novel VUMs achieved simulation performances closer to those of the real users. With a better VUM quality available, the last study was performed on a real world interface problem concerning the interface accessibility assessment for GUI designers. The Metaverse designers redesigned its interface so as not to exclude MCI users from the potential body of target users. The new design was evaluated by the novel VUMs to indicate results according to which, when the MCI cognitive submodule was enabled and active, the simulator produced results closer to the real MCI users, especially in memory-demanding and decision making tasks.

The followed user-centric methodology and mainly the novel VUMs created by this study can be used in most cases of interface accessibility assessment covering over 90% of all common interface accessibility issues. It is limited only by an extreme cognitive disability threshold which corresponds to the 90% MCI users. VUMs of very high dementia levels can produce valid simulation results, but their duration predictions cannot always be valuable for interface designers as extreme interventions on an interface design may cause the interface to lose an important part of its functionality.

## 8. Conclusion

Commonly, GUI designers rescale GUI elements or increase color contrast to serve the needs of elderly or impaired users. However, there is still need for specific interface design strategy for the reconfiguration and change of visual metaphors to customize interfaces to the specific needs of groups with cognitive related limitations. Our approach combined literature findings and experimental data to predict in better accuracy the performance of cognitive impaired VUMs over known interface accessibility limitations. In the proposed VUM architecture implementation, we inserted an additional cognitive submodule to produce “noisy” functionality in the internal VUM structure instead of producing noisy data in the output.

Pilot tests on the simulator indicated high correlation between the tests scores of real users and VUMs. These findings should be viewed as an early approach about the latent interest in encompassing cognitive features in the design of infotainment simulation models. Evaluation tests from infotainment products have shown that the proposed VUMs' modification customized according to cognitive impairments can be a valuable asset to the designer, as a way of increasing products' and services accessibility for elderly with varying deficits of memory, attention, judgment, and communication ability. The utilised scenarios required not only memory but even more complex cognitive functions, such as decision making abilities.

After the encouraging results extracted from the pilot test presented herein, the forthcoming tests will be performed by recruiting a wider body of subjects (30–50 persons). Those tests will include the test-retest reliability evaluation of the computer-based screening test and, moreover, it is expected to improve the prediction accuracy of the simulation results between VUMs and actual users. Recruitment criteria could be extended so as to include a depression detection test like the Beck Depression Inventory (BDI-II) to eliminate possible effects caused by extraneous to the MCI itself factors. Moreover, among our future plans is the actual use and evaluation of vocal and aural modules.

This research effort, being in-line with the “design for all” principle, is helping people with cognitive functionality decline to improve their quality of life in an indirect way: not by providing therapeutic appliances straight to them, but with changing the design culture of the industrial world on their behalf.

## Figures and Tables

**Table 1:** MCI screening test results.

Variables	Young	Healthy elderly	MCI
Sex M/F	4/7	10/5	6/4
Age (in years)	26.27 (SD = 1.95)	64.69 (SD = 4.80)	66.70 (SD = 7.18)
Education (in years)	17.36 (SD = 0.80)	13.46 (SD = 3.33)	12.9 (SD = 3.38)
MoCa number of words (in 60 sec)	13.64 (SD = 4.03)	13.80 (SD = 4.94)	9.1 (SD = 4.84)
MoCa duration between words (in sec)	4.70 (SD = 1.186)	5.05 (SD = 2.36)	8.45 (SD = 4.76)
BNT answers without help	25.56 (SD = 2.18)	25.90 (SD = 1.97)	21.78 (SD = 5.40)
BNT answers with semantic help	26.00 (SD = 2.06)	26.20 (SD = 1.39)	22.22 (SD = 5.12)
BNT answers with phonemic help	26.11 (SD = 2.14)	26.20 (SD = 1.39)	22.56 (SD = 5)
BNT duration (in sec)	113.3 (SD = 33.60)	117.49 (SD = 21.80)	167.3 (SD = 71.65)

**Table 2:** Scenarios performed in the infotainment pilot test.

id	Scenario name	Scenario description	Tasks	Required abilities
S1	Enter the Metaverse	It is required that users type username and password	5	Memory
S2	Change Outfit	Having a second outfit available, users are asked to change from outfit 1 to outfit 2	5	Decision-perception
S3	Upload file in Metaverse	Choose an image file from the local drive and upload	8	Information orientation
S4	Build 3D Object	Create a new cube in the virtual environment	5	Perception-reflection
S5	Scale 3D Object	Scale the cube to make its side equal to 1 m	5	Perception-reflection
S6	Rotate 3D Object	Rotate the cube in *x*, *y*, and *z* using the colored rotation rings	8	Motor-vision
S7	Move 3D Object	Move the 3D object in 3 directions using the colored moving arrows	8	Motor-vision
S8	Navigate Avatar in Free Mode	Rotate the head-camera of the avatar in space and then move few steps forward	3	Visual-motor
S9	Navigate Avatar to Sound Source	Navigate the avatar from a random position to the source of the sound	4	Visual-acoustic-motor-decision-perception
S10	Interact with Dynamic Object	Touch an object with dynamic behaviour	2	Motor-perception
S11	Interact with Multimedia Object	Touch an object which makes a sound	2	Motor-perception
S12	Initiate Chat with Another User	Locate another user in the Metaverse and send a “Hello” message	5	Perception-verbal
S13	Share Folder with Another User	Share folder with another user	9	Information orientation-memory

**Table 3:** Performed scenarios and scores.

Scenario	Optimal user		Healthy elderly user		MCI User	
	Events	Time^*^	Events	Time^*^ (SD)	Events	Time^*^ (SD)
S1	46	15.35	59.82 (23.43)	59.53 (21.85)	53.63 (5.07)	87.00 (32.34)
S2	10	8.73	13.64 (2.65)	46.88 (19.07)	15.00 (3.20)	56.75 (15.55)
S3	16	15.65	26.18 (9.44)	104.180 (44.35)	24.25 (8.37)	126.54 (44.07)
S4	10	6.64	11.91 (2.70)	35.37 (16.93)	13.38 (2.56)	47.33 (21.91)
S5	10	12.82	15.64 (7.31)	55.65 (32.80)	17.00 (3.38)	73.65 (23.19)
S6	12	12.15	20.36 (4.34)	63.3 (19.98)	20.88 (7.08)	63.50 (18.54)
S7	12	15.36	19.82 (4.99)	54.08 (19.75)	28.50 (16.41)	69.78 (25.901)
S8	6	4.59	7.82 (2.60)	14.07 (6.33)	6.88 (1.24)	16.93 (7.09)
S9	12	7.39	39.36 (25.31)	42.10 (16.24)	23.63 (9.70)	30.73 (15.55)
S10	4	1.51	5.09 (1.86)	10.10 (6.54)	5.38 (2.66)	12.36 (7.80)
S11	4	1.03	4.45 (0.82)	5.20 (3.80)	5.57 (4.20)	7.01 (7.42)
S12	22	12.79	26.64 (8.60)	51.97 (23.00)	26.13 (9.07)	47.68 (10.77)
S13	27	14.86	33.64 (10.14)	83.43 (25.05)	42.50 (23.39)	121.53 (45.42)

^*^Time is in seconds.

**Table 4:** Test the new VUMs by example.

Users	Comments	S1 duration in sec	Fraction of the average MCI user
OpUs	Duration of the optimal user	15.35	0.17
Healthy Elderly	Score recorded in the infotainment pilot test	59.53	0.68
MCI	Score recorded by the actual MCI users	87.00	1.00
50Elderly	VUM of the first generation	17.30	0.19
50MCI	VUM of the second generation (MCI-ready)	78.38	0.90
